# Purchasing “Nootropics” Online: Identification and Quantification of Ingredients in Phenibut-Containing Products

**DOI:** 10.3390/medicina60101561

**Published:** 2024-09-24

**Authors:** Toms Upmanis, Eduards Sevostjanovs, Liga Zvejniece, Helena Kazoka, Vadims Kisis, Osvalds Pugovics, Maija Dambrova

**Affiliations:** 1 Laboratory of Chromatography, Latvian Institute of Organic Synthesis, LV-1006 Riga, Latvia; upmanis@osi.lv (T.U.); helena@osi.lv (H.K.); 2 Laboratory of Physical Organic Chemistry, Latvian Institute of Organic Synthesis, LV-1006 Riga, Latvia; eduards@osi.lv; 3 Laboratory of Pharmaceutical Pharmacology, Latvian Institute of Organic Synthesis, LV-1006 Riga, Latvia; liga@farm.osi.lv; 4 Medical and Clinical Research Department, JSC Olpha, LV-2114 Olaine, Latvia; Vadims.Kisis@olpha.eu; 5 Latvian Institute of Organic Synthesis, LV-1006 Riga, Latvia; osvalds@osi.lv; 6 Department of Pharmaceutical Chemistry, Riga Stradins University, LV-1007 Riga, Latvia

**Keywords:** phenibut, online-purchased drugs, pharmaceutical analysis, HPLC–DAD, LC–MS

## Abstract

*Background and Objectives*: Phenibut is a central nervous system drug that is registered and used in clinical practice as a prescription medication. In recent decades, the drug has become popular as a “nootropic and cognition enhancer” because of its active marketing as a dietary or food supplement sold online. This has resulted in a growing number of case reports on acute toxicity and withdrawal symptoms and has raised concerns about the quality of phenibut-containing products. *Materials and Methods*: We used high-performance liquid chromatography with diode-array detection and ultra-performance liquid chromatography–mass spectrometry to investigate the quality of six phenibut-containing samples purchased from three internet suppliers. *Results*: Substantially lower levels of the active pharmaceutical ingredient than claimed on the packaging were found for three of the supplements tested. A considerably higher level of phenibut was present in one product. All online-purchased phenibut-containing capsules included declared and undeclared ingredients found in various dietary supplements claiming to have metabolic, neurotropic, antioxidant, anti-inflammatory, and neuroprotective properties. *Conclusions*: The online-purchased phenibut products contained undeclared ingredients and the content of phenibut differed from the declared. The combinations of these additional ingredients with phenibut have not been tested for activity or safety and their use warrants further attention to avoid potential health problems.

## 1. Introduction

**Figure 1:**
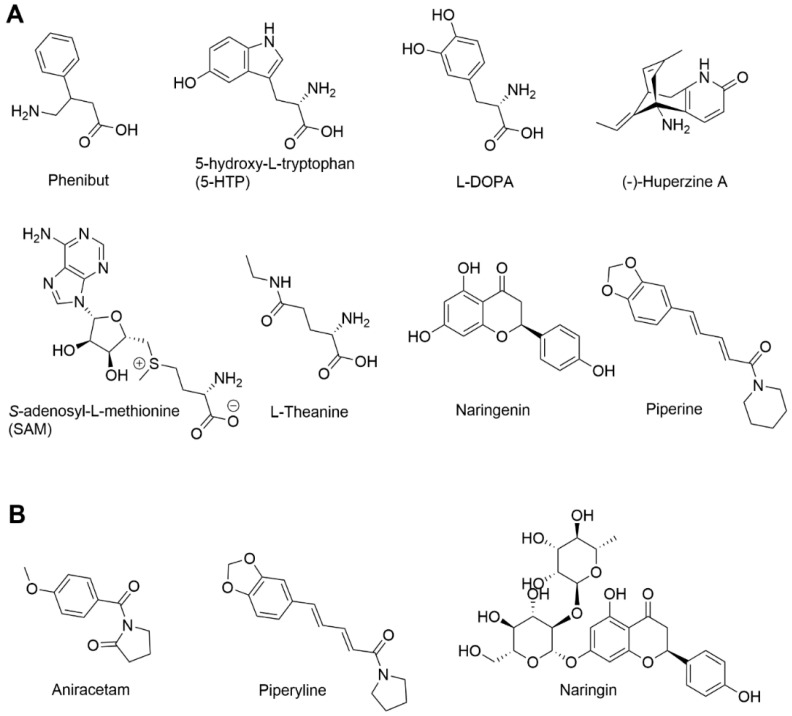
Structures of compounds found in online-purchased phenibut-containing products within this study: (**A**) declared by manufacturers; (**B**) undeclared compounds.

The over-the-counter purchase of drugs that are typically distributed through prescription, as well as the purity and quality of such pharmaceutical products, is a growing global problem. Phenibut (PHB, Fenibut, Noofen, Anvifen) is a central nervous system (CNS) drug that is registered and used in clinical practice in Eastern European countries as a prescription medication for the treatment of anxiety, tics, stuttering, insomnia, dizziness, and alcohol abstinence [1,2]. Structurally, phenibut is a phenyl derivative of the neurotransmitter γ-aminobutyric acid (GABA) (Figure 1A). This drug is usually produced as a racemic mixture of two optical isomers, of which only R-phenibut ((3R)-phenyl-4-aminobutyric acid) has a binding affinity for gamma-aminobutyric acid B (GABA-B) receptors, while both R- and S-phenibut bind to the α_2_δ subunit of voltage-dependent calcium channels (VDCCs) [3,4,5]. The clinically approved pharmacological effects of phenibut can be explained by its interaction with these drug targets.

In recent decades, phenibut has become popular as a “nootropic and cognition enhancer” because of its active marketing as a dietary or food supplement sold online. The United States Food and Drug Administration (FDA) has warned that the drug should not be permitted in over-the-counter supplements, but some producers continued to include and even increased the quantity of phenibut in their products, with the dose of phenibut ranging from 21 mg to 1164 mg per serving [6]. This has resulted in the uncontrolled use of the substance at gram-scale doses and a growing number of case reports on its acute toxicity and withdrawal symptoms [7,8]. However, phenibut dependence and intoxication cases have been reported mostly for the cases of its co-ingestion with alcohol [9] and other substances with recreational psychotropic properties, in patients having coexisting substance abuse disorders to other drugs [10]. In addition, the purity and quality of the consumed phenibut-containing product probably remained questionable and therefore was not reported.

This study aimed both to quantify the actual content of the active pharmaceutical ingredient (API) phenibut and to investigate the potential addition of undeclared adulterants (Figure 1B) in online-purchased phenibut-containing products. To our knowledge, this is the first study both identifying and quantifying undeclared ingredients in the phenibut-containing over-the-counter products. Liquid chromatography hyphenated to high-resolution mass spectrometry (UHPLC–TOF–MS) method and high-performance liquid chromatography with diode array detection (HPLC–DAD) methods were developed to identify and quantify the content of both active ingredients declared ingredients (Figure 1A) and undeclared adulterants (Figure 1B). The identification of undeclared ingredients was made by the calculation of molecular formulae from UHPLC–TOF–MS data followed by confirmation of retention times against reference standards by the HPLC–DAD method.

## 2. Materials and Methods

### 2.1. Sampling

This study was conducted on six phenibut samples purchased from three internet suppliers (Figure S1) between June 2023 and September 2023. The four samples were labeled with the term “dietary supplement”, but two other samples did not have such statements. All products (100%) were capsules, from which one (16.6%) was vegetable gelatin capsules, one (16.6%) was hydroxypropylmethylcellulose capsules, and four remaining (66.8%)—a nondeclared type of capsule (Table S1). A pool of 20 capsules was prepared by emptying and accurately weighing the contents of twenty capsules from each online-purchased phenibut sample. The contents of the capsules were ground into a fine powder using a mortar and pestle, mixed thoroughly, and used for the sample preparation in all further analytical experiments.

### 2.2. Instrumentation and Apparatus

High-performance liquid chromatography with diode array detection (HPLC–DAD) was used to analyze the phenibut content in six different products marketed as nootropics. Other pharmaceutically active compounds in these supplements were tentatively identified by ultrahigh-performance liquid chromatography–time of flight mass spectrometry (UHPLC–TOF–MS) and further quantified by HPLC–DAD (see Supplementary Information). UHPLC–TOF–MS analysis was performed using a Shimadzu LCMS–IT–TOF (Shimadzu Corporation, Kyoto, Japan) liquid chromatograph mass spectrometer, consisting of a Nexera X2 LC30 series chromatography system coupled to a hybrid ion trap/time-of-flight mass spectrometer. The instrument was operated and data were processed using the Shimadzu LCMSsolution Ver3 software (Shimadzu Corporation, Kyoto, Japan). HPLC–DAD analyses were performed on a Waters Alliance (Waters Corporation, Milford, MA, USA) instrument equipped with an e2695 separations module, consisting of a quaternary pump, degasser, autosampler, and column heater. A Waters 2998 photodiode array detector was used for the detection of analytes. The output signal was monitored and processed using Waters Empower 3 software (Waters Corporation, Milford, MA, USA), as described in the Supplementary Information.

## 3. Results

### 3.1. Visual Inspection

All of the tested phenibut-containing products were manufactured in the USA and sold in capsule form. In four of the tested products, the type of capsule was not declared, but the two remaining products were in vegetable gelatine and hydroxypropylmethyl-cellulose capsules. The product capsule size and images of the emptied contents are presented in Figure S1 and Table S1. Visual inspection of the samples revealed the first inconsistencies between the declared and actual compositions in products 3 and 5, where the declared weight of phenibut in product 5 and the weight of all declared components in the product 3 formulation (Table 1 and Table 2) exceeded the experimentally determined mass of the emptied capsule contents, even without considering the presence of the stated excipients (magnesium stearate and silicon dioxide).

### 3.2. Quantification of the Active Pharmaceutical Ingredient—Phenibut

An HPLC–DAD method was used for the separation and quantification of the listed active pharmaceutical ingredient. The presence of phenibut was confirmed in all six samples (Table 1, Figure S2). Samples obtained from products 1 and 3 had the closest phenibut contents to the declared contents (490 mg and 524 mg, respectively, of the 500 mg declared). Substantially lower levels of API than claimed on the packaging were found for three of the supplements tested: on average, 113 mg or less than half of the declared 250 mg phenibut content was found in product 2, 385 mg instead of 500 mg phenibut was found in product 4, and 689 mg of phenibut was found in product 5 (900 mg claimed on the packaging). Considerably higher levels of the API were present in product six samples, where an average of 668 mg of phenibut was found instead of 500 mg. 

### 3.3. Identification of Other Ingredients in Online-Purchased Phenibut Products

The presence of other added pharmaceutically active components (Figure 1) was confirmed by a UHPLC–TOF–MS method using extracted ion chromatograms (XICs) by specifying the *m*/*z* of the protonated molecular ion [M+H]^+^ for each component. Five out of seven declared pharmaceutically active components were detected (Table 2). Three other compounds were observed in the total ion chromatograms (TICs). Using elemental composition calculations of the observed mass spectra, the peaks were tentatively identified as aniracetam, piperyline, and naringin. The results are summarized in Table 2. In addition to the presence of undeclared components, the contents of the declared components were not consistent with the specifications of any of the tested phenibut-containing products. Phenibut products 1 and 2 were sold as supplements containing no other pharmaceutically active ingredients. However, in both samples, a peak with *m*/*z* 220.0963 was observed at a retention time of 7.24 min (Figure S3). Elemental composition calculations revealed that the peak was C_12_H_13_NO_3_ with a mass error of 0.5 mDa, which was tentatively identified as aniracetam. The peak at a retention time of 6.80 min was observed in the total ion chromatograms of phenibut samples 3, 5, and 6. Figure S4A shows chromatograms of phenibut product 3. The measured *m*/*z* in product 3 was 581.1839 (Figure S4B).

The spectrum also contains a peak with *m*/*z* 273.0736, which corresponds to naringenin. The elemental composition calculation of *m*/*z* 581.1839 led to a formula of C_27_H_32_O_14_ with a mass error of 2.6 mDa. The peak was assumed to be of naringin, a diglycoside of naringenin. The use of free naringenin as an additive was declared only in sample 6, where it was found in the form of diglycoside naringin. Free naringenin was detected in sample 4 (Figure S5A,B), where no other ingredients were listed on the label. In the total ion chromatograms of the phenibut-containing products 3, 4, 5, and 6, a peak at a retention time of 10.36 min was observed. The measured *m*/*z* in product 4 was 272.1269 (Figure S5C). The elemental composition calculation gives the formula C_16_H_17_NO_3_ with a mass error of 1.2 mDa. The peak was tentatively identified as piperyline, a homologue of piperine.

The compounds identified by UHPLC-TOF–MS were then quantified by HPLC–DAD, and the corresponding results are summarized in Table 2. In general, the composition of none of the studied supplements was consistent with the manufacturer’s specifications. From the listed ingredients in products 3 and 6 (Table 1), no traces of huperzine-A (Figure S6) or S-(5′-adenosyl)-L-methionine (SAM; Figure S7) were found in any of the studied samples. Overall, by comparing the amount of phenibut found and the impurity profile in products 5 and 6, it can be concluded that both of these products likely contain the same formulation of ingredients, even though they are sold under different brand names and are marketed as different products—product 5 as pure phenibut and 6 as a formulation of various active compounds. Based on the composition of product 3, it is very likely that the formulation for this product is also supplied from the same source, and differences in the measured concentrations of individual ingredients are related to the amount of added excipients during capsule preparation. All three of these products contained 5-HTP (Figure S8), L-DOPA (Figure S9), L-theanine (Figure S10), and piperine (Figure S11) at substantially lower levels (Table 2) than claimed on the packaging. Only trace amounts of naringenin (Figure S12) were found in the capsules of phenibut-containing products 3, 5, and 6. However, low amounts of the diglycoside naringin were found in these three products. A different composition of other added active ingredients was detected in product 4, which was originally marketed as pure phenibut; however, it contained the highest amounts among the studied supplements of L-DOPA (Figure S9), L-theanine (Figure S10) and naringenin (Figure S12) and lower levels of piperine (Figure S11).

Among the undeclared ingredients, low amounts of aniracetam, an average of 0.80 and 0.28 mg per capsule (Figure S13), were detected in the samples of products 1 and 2, respectively. Another undeclared ingredient, piperyline, was found in the samples of supplements 3, 4, 5, and 6 (Figure S14).

## 4. Discussion

Phenibut is a CNS drug prescribed to treat neurological disorders [1,2]. During the last decade, significant concerns have been raised about the widespread availability of the drug as a dietary or food supplement sold online [6,11]. It has been already reported that phenibut-containing supplements contain higher doses than recommended [2,6]; however, other ingredients in the online-purchased phenibut products had not been analyzed before. 

The combination of HPLC–DAD and UHPLC–TOF–MS turned out to be a powerful tool for identifying and quantifying undeclared ingredients in online-purchased products. The declared and undeclared compounds found in online-purchased phenibut-containing capsules (Figure 1) included ingredients found in various “nootropic” dietary supplements [12] that have claimed to have metabolic (5-HTP, piperine); neurotropic (5-HTP, L-DOPA, L-theanine, aniracetam); and antioxidant, anti-inflammatory and neuroprotective properties (naringenin, naringin, huperzine-A) [13,14]. The combinations of these additional ingredients with phenibut have not been tested for activity or safety and their use also might raise health concerns. Excessive dosing of phenibut and potentially synergistic combinations with emerging recreational supplements warrant further attention in future studies of “nootropic” dietary products.

The content of declared and undeclared ingredients in the online-purchased phenibut-containing products was relatively low (Table 2) to induce any therapeutic effect. For example, the dose of aniracetam required to achieve the desired pharmaceutical effect ranges from 10 to 100 mg/kg [15,16]; therefore, it is unlikely that the low levels of this drug found in products 1 and 2 were intentionally added, and the source of aniracetam is likely to be related to cross-contamination at the capsule filling stage. One of the amide alkaloids isolated from black peppers [17], piperyline, found in products 3, 4, 5, and 6 is likely an impurity in poorly standardized black pepper extract which is a common manufacturing issue of food supplements [18]. 

Our study found that all online-purchased phenibut-containing products contained undeclared ingredients (Table 2). In addition, we found that the content of declared ingredients also differed remarkably, and was both lower and higher than indicated. For example, the content of phenibut was just 45% of the declared in product 2, while its content in product 6 was 134% (Table 1). The cases of phenibut dependence and intoxication have reported daily doses that exceeded the recommended dose by ~4- to 10-fold [2,19], reaching even 14 and 100 g [20,21]. The data of our study show that most of the products contained less phenibut than declared. However, the highest recommended daily doses suggested by several manufacturers in the formulation sheets reach several grams daily (3.6 and 4 g, Table 1) and considerably exceed therapeutic doses (from 250 to 2000 mg). In three of the tested products, the recommended dose also turned out to be >2 g after correcting the producer-recommended doses according to API content found in products. These data show that in cases of intoxication with online-purchased phenibut products, the real amount of consumed phenibut remains unknown unless analyzed in each case. 

## 5. Conclusions

The online-purchased phenibut products contained undeclared ingredients and the content of phenibut differed from the declared. The combinations of these additional ingredients with phenibut have not been tested for activity or safety, should not be sold as food supplements without prescription, and warrant further investigation to avoid potential health problems.

## Figures and Tables

**Table 1:** Characteristics of the tested phenibut products.

Samples	Brand Name ^a^	API Labelled, mg	API Found, Mean ± SD, mg ^b^	Capsule Contents, Mean ± SD, mg ^c^	Other Listed Ingredients (without Excipients)	Recommended Dose, mg ^d^	Corrected Dose, mg ^e^
1	Phenibut HCl	500	490 ± 3.5	910 ± 6.8		-	-
2	PHENIBUT	250	113 ± 2.0	381 ± 15		-	-
3	PHENI-B ULTRA	500	524 ± 2.2	630 ± 2.0	5-HTP, l-DOPA l-theanine huperzine-A	1500	1572
4	PHENIBUT	500	385 ± 3.1	624 ± 7.9		4000	3080
5	PHENIBUT^RX^	900	689 ± 4.3	897 ± 32		3600	2756
6	PHENI+	500	668 ± 8.8	855 ± 41	5-HTP, l-theanine SAM, piperine naringenin, huperzine-A	1500	2004

List of product brand names, API contents, other listed ingredients, phenibut contents, and recommended doses in online-purchased phenibut supplements. Abbreviations: active pharmaceutical ingredient, API; standard deviation, SD; S-adenosyl-l-methionine, SAM; 5-hydroxy-l-tryptophan, 5-HTP; l-3,4-dihydroxyphenylalanine, l-DOPA. ^a^ Manufacturers are shown in Figure S1; ^b^ n = 3; ^c^ n = 20; ^d^ Highest recommended daily dose suggested by the manufacturer in the formulation sheet (Table S2); ^e^ Corrected values according to API found in products; “-”: no data provided.

**Table 2:** Other ingredients detected in online-purchased phenibut-containing products.

Compound/ Chemical Formula/ Calculated *m*/*z* [M+H]^+^	Other Ingredients Found, Mean ± SD, mg (Declared, mg) Per Capsule					
	1	2	3	4	5	6
5-HTP: C_11_H_12_N_2_O_3_ 221.0921			16.9 ± 0.4 (50)		21.5 ± 0.8 Not declared	20.9 ± 0.2 (50)
l-DOPA: C_9_H_11_NO_4_ 198.0761			16.7 ± 0.4 (50)	68.8 ± 0.4 Not declared	22.0 ± 2.8 Not declared	23.0 ± 1.9 Not declared
l-Theanine: C_7_H_14_N_2_O_3_ 175.1077			16.3 ± 0.2 (50)	93.4 ± 1.1 Not declared	21.2 ± 0.7 Not declared	20.2 ± 0.4 (100)
Piperine: C_17_H_19_NO_3_ 286.1438			5.7 ± 0.2 Not declared	3.2 ± 0.1 Not declared	7.6 ± 0.7 Not declared	7.5 ± 0.2 (10)
Naringenin: C_15_H_12_O_5_ 273.0758			0.012 ± 0.002 Not declared	3.0 ± 0.1 Not declared	0.015 ± 0.001 Not declared	0.014 ± 0.001 (10)
SAM: C_15_H_22_N_6_O_5_S 399.1445						- (100)
Huperzine-A: C_15_H_18_N_2_O 243.1492			- (0.1)			- (0.1)
Piperyline: C_16_H_17_NO_3_ 272.1281			0.08 ± 0.002 Not declared	0.03 ± 0.002 Not declared	0.11 ± 0.01 Not declared	0.11 ± 0.002 Not declared
Naringin: C_27_H_32_0_14_ 581.1865			2.8 ± 0.1 Not declared		3.8 ± 0.2 Not declared	3.7 ± 0.03 Not declared
Aniracetam: C_12_H_13_NO_3_ 220.0968	0.80 ± 0.03 Not declared	0.28 ± 0.01 Not declared				

Abbreviations: 5-hydroxy-l-tryptophan, 5-HTP; l-3,4-dihydroxyphenylalanine, l-DOPA; *S*-adenosyl-l-methionine, SAM; not detected, “-”.

## Data Availability

The raw data supporting the conclusions of this article will be made available by the authors on request.

## Supplementary Materials

The following supporting information can be downloaded at: https://www.mdpi.com/article/10.3390/medicina60101561/s1, Table S1: Online-purchased phenibut product capsule excipients; Table S2: The manufacturer-provided information on the use of online-purchased phenibut products; Figure S1: Visual inspection of online-purchased phenibut products; Figure S2: HPLC–DAD analysis of phenibut contents in online-purchased products; Figure S3: UHPLC–TOF–MS analysis of sample 1; Figure S4: UHPLC–TOF–MS analysis of sample 3; Figure S5: UHPLC–TOF–MS analysis of sample 4; Figure S6: Huperzine A HPLC–DAD analysis in online-purchased phenibut products; Figure S7: S-(5′-Adenosyl)-L-methionine (SAM) HPLC–DAD analysis in online-purchased phenibut products; Figure S8: 5-Hydroxy-L-tryptophan (5-HTP) HPLC–DAD analysis in online-purchased phenibut products; Figure S9: L-3,4-Dihydroxyphenylalanine (L-DOPA) HPLC–DAD analysis in online-purchased phenibut products; Figure S10: L-Theanine HPLC–DAD analysis in online-purchased phenibut products; Figure S11: Piperine HPLC–DAD analysis in online-purchased phenibut products; Figure S12: Naringenin and naringin HPLC–DAD analysis in online-purchased phenibut products; Figure S13: Aniracetam HPLC–DAD analysis in online-purchased phenibut products; Figure S14: Piperyline HPLC–DAD analysis in online-purchased phenibut products.
