# Supplementary material for: Purchasing “Nootropics” Online: Identification and Quantification of Ingredients in Phenibut-Containing Products
Source: Medicina (Kaunas). 2024 Sep 24;60(10):1561. doi: 10.3390/medicina60101561 (PMC11509446; doi:10.3390/medicina60101561)
Supplement: Supplementary file 1 [file medicina-60-01561-s001.zip › medicina-3158069-supplementary.pdf]

Supplementary information

Purchasing "Nootropics" Online: Identification and Quantification of Ingredients in Phenibut-Containing Products

Toms Upmanis [1], Eduards Sevostjanovs [2], Liga Zvejniece [3], Helena Kazoka [1], Vadims Kisis [4], Osvalds Pugovics [5] and Maija Dambrova [3,6,*]

[1] Laboratory of Chromatography, Latvian Institute of Organic Synthesis, Riga LV-1006, Latvia
[2] Laboratory of Physical Organic Chemistry, Latvian Institute of Organic Synthesis, Riga LV-1006, Latvia
[3] Laboratory of Pharmaceutical Pharmacology, Latvian Institute of Organic Synthesis, Riga LV-1006, Latvia
[4] Medical and Clinical Research Department, JSC Olpha, Olaine LV-2114, Latvia
[5] Latvian Institute of Organic Synthesis, Riga LV-1006, Latvia
[6] Department of Pharmaceutical Chemistry, Riga Stradins University, Riga LV-1007, Latvia
* Correspondence: maija.dambrova@farm.osi.lv

Table of contents

Table of contents 2

1. Materials and methods 3

    1.1. Sampling 3

    1.2. Instrumentation and apparatus 3

        1.2.1. UHPLC-TOF-MS 3

        1.2.3. HPLC-DAD 3

    1.3. Standards and reagents 3

    1.4. Assay of phenibut content in online purchased phenibut product capsules 4

    1.5. UHPLC-TOF-MS analysis 4

    1.6. Identification and specificity assay of other ingredients in online purchased phenibut supplement capsules 4

2. Tables 7

    Table S1.. 7

    Table S2. 8

3. Figures 11

    Figure S1. 11

    Figure S2. 12

    Figure S3. 13

    Figure S4. 14

    Figure S5. 15

    Figure S6. 16

    Figure S7. 17

    Figure S8. 18

    Figure S9. 19

    Figure S10. 20

    Figure S11. 21

    Figure S12. 22

    Figure S13. 23

    Figure S14. 24

1. Materials and methods

1.1. Sampling

The study was conducted on six phenibut samples purchased from three internet suppliers (www.stay-focused.com, www.nextvalley.com, and www.paradigmpeptides.com) between June 2023 and September 2023. The label of four samples presented the term "dietary supplement", but two other samples obstained from such a statement. All of the phenibut samples obtained for this study were manufactured in the USA. Regarding the dosage forms, all products (100%) were capsules, from which one (16.6%) was vegetable gelatin capsules, 1 (16.6%) was hydroxypropylmethylcellulose capsules, and four remaining (66.8%) - a nondeclared type of capsule (Table S1). A pool of 20 capsules was prepared by emptying and accurately weighing the contents of twenty capsules from each online purchased phenibut sample. The contents of the capsules were ground into a fine powder using a mortar and pestle, mixed thoroughly, and used for the sample preparation in all further analytical experiments.

1.2. Instrumentation and apparatus

1.2.1. UHPLC-TOF-MS

The ultra-high performance liquid chromatography - time of flight mass spectrometry (UHPLC-TOF-MS) analysis was performed on Shimadzu LCMS-IT-TOF (Shimadzu Corporation, Kyoto, Japan) liquid chromatograph mass spectrometer, consisting of Nexera X2 LC30 series chromatography system coupled to hybrid ion trap/time-of-flight mass spectrometer. The instrument was operated and data were processed using the Shimadzu LCMSsolution Ver3 software (Shimadzu Corporation, Kyoto, Japan).

1.2.3. HPLC-DAD

The chromatographic analysis was performed on Waters Alliance (Waters Corporation, Milford, MA, USA) instrument equipped with an e2695 separations module, consisting of a quaternary pump, degasser, autosampler, and column heater. Waters 2998 photodiode array detector was used for the detection of analytes. The output signal was monitored and processed using Waters Empower 3 software (Waters Corporation, Milford, MA, USA).

1.3. Standards and reagents

Phenibut·HCl working standard was provided by JSC Olpha (Olaine, Latvia). Certified reference standard of L-3,4-dihydroxyphenylalanine (L-DOPA; 99.7%) and analytical standards of 5-hydroxy-L-trypthophan (5-HTP; ≥ 98%), (-)-huperzine A (≥ 98%), piperine (≥ 97%), (±)-naringenin (≥ 95%), L-theanine (> 98.0%) and aniracetam (≥ 99%) were purchased from Merck (Darmstadt, Germany). Analytical standard of *S*-(5'-Adenosyl)-L-methionine dihydrochloride (SAM; ≥ 95%) was purchased from Cayman Chemical (Ann Arbor, MI, USA) and piperyline (99.7%) from AmBeed (Arlington Heights, IL, USA).

For HPLC analysis, gradient grade acetonitrile (ACN), methanol (MeOH), and heptane-1-sulfonic acid sodium salt were obtained from Merck (Darmstadt, Germany). Gradient-grade deionized water (R ≥ 18 MΩ·cm, total organic carbon (TOC) concentration ≤ 3 ppb) was produced by the Milli-Q system (Millipore, Darmstadt, Germany). HPLC grade orthophosphoric acid (85%-90%) was purchased

from Fluka (Buchs, Switzerland), HPLC grade trifluoroacetic acid (99%), and potassium dihydrogen orthophosphate (≥ 99%) were obtained from Fisher Chemicals (Loughborough, UK).

For mass spectrometry analysis, hypergrade LC-MS acetonitrile was obtained from Merck (Darmstadt, Germany). LC-MS grade formic acid was from Honeywell (Seelze, Germany).

1.4. Assay of phenibut content in online purchased phenibut product capsules

The chromatographic analysis was performed by in-house HPLC method. Separations were carried out using YMC-Pack Pro C_8 column (4.6 mm × 150 mm, 5 µm; YMC Europe Gmbh, Dinslaken, Germany). Column temperature was maintained at 30°C with a flow rate of 1 mL/min. For the mobile phase, acetonitrile:0.02M phosphate buffer (pH 2.8) (2:98 *V/V*) - eluent A; and (70:30 *V/V*) - eluent B were used. The linear gradient mode used for the elution was as follows: 0-25 min, 0%-50% B; 25-30 min, 50% B; 30-33 min, 50%-0% B; 33-40 min 0% B. The UV detection was performed at 210 nm and injection volume was set to 25 µL.

Phenibut stock solution was prepared by dissolving 200 mg phenibut·HCl working standard into 100 mL of eluent A. The reference solution was then prepared by 100-fold dilution of the phenibut stock solution with eluent A, resulting in c_{sample} = 0.02 mg/mL.

Accurate weights of capsule powder equivalent to 200 mg phenibut·HCl of each online purchased phenibut product (Table 1 in the article) were extracted in 100 mL of eluent A (in triplicate) with the aid of sonication for 10 minutes at 40°C to obtain respective stock solutions. The suspensions were then filtered through a PTFE filter with a pore diameter of 0.45 µm and the filtrates were diluted 100-fold, resulting in analytical sample solutions used for HPLC-DAD assay and LC-TOF-MS profiling analysis.

Active pharmaceutical ingredient (API) content in online purchased product capsules was determined by plotting the mean peak area of phenibut in analytical sample solutions against the mean peak area in the reference solution.

1.5. UHPLC-TOF-MS analysis

The chromatographic separation was performed using Waters Acquity UPLC BEH C_{18} column (2.1 mm × 50 mm, 1.7 µm; Waters Corporation, Milford, MA, USA). Column temperature was maintained at 40°C with a flow rate of 0.4 mL/min. For the mobile phase, 0.1% formic acid in water – eluent A; and acetonitrile – eluent B were used. The linear gradient mode used for the elution was as follows: 0-15 min, 0.5%-98% B; 15-25 min, 98% B; 25-26 min, 98%-0.5% B; 26-30 min, 0.5% B. Injection volume was set to 10 µL.

The mass spectrometer was operated in electrospray ionization (ESI) mode. The capillary voltage was 4.5 kV in positive ionization and -3.5 kV in negative ionization. The heat block temperature was 200°C, CDL temperature was 200°C, nebulizing gas flow was 1.5 L/min, detector voltage was 1.5 kV, ion accumulation time was 10 msec and the mass range was 150 – 1000 Da.

1.6. Identification and specificity assay of other ingredients in online purchased phenibut supplement capsules

For L-DOPA, 5-HTP, and SAM the chromatographic analyses were performed on the Apollo C_{18} column (4.6 mm × 150 mm, 5 µm; Grace, Carnforth, United Kingdom). Column temperature was

maintained at 40°C with a flow rate of 1 mL/min. For the mobile phase, a mixture of methanol (eluent B) and 0.2% trifluoroacetic acid in water (eluent A) was used. The linear gradient mode used for the elution was as follows: 0-15 min, 5%-95% B; 15-20 min, 95% B; 20-22 min, 95%-5% B; 22-25 min 5% B. The UV detection was performed at 254 nm (SAM) and 275 nm (5-HTP; L-DOPA) and the injection volume was set to 25 μL.

L-DOPA and 5-HTP reference solutions were prepared by dissolving 5 mg of standards into 10 mL of methanol: water: trifluoracetic acid (5:94.8:0.2 *V*/*V*/*V*) - diluent 1 and diluting the obtained stock solutions 10-fold (c_{sample} = 0.05 mg/mL).

SAM reference solution was prepared by dissolving 2.5 mg of the standard into 1 mL of diluent 1 and diluting the obtained stock solution 2000-fold (c_{sample} = 0.00125 mg/mL).

For huperzine A, naringenin, piperine, piperyline, and aniracetam, the chromatographic analyses were performed on the Apollo C_{18} column (4.6 mm × 150 mm, 5 μm; HiChrom, Berkshire, United Kingdom). Column temperature was maintained at 40°C with a flow rate of 1 mL/min. For the mobile phase, a mixture of acetonitrile (eluent B) with 0.1% orthophosphoric acid in water (eluent A) was used. The linear gradient mode used for the elution was as follows: 0-15 min, 5%-95% B; 15-20 min, 95% B; 20-22 min, 95%-5% B; 22-25 min 5% B. The UV detection was performed at 308 nm (huperzine A, piperine, and piperyline) and 280 nm (naringenin and aniracetam), and the injection volume was set to 25 μL.

Huperzine A reference solution was prepared by dissolving 10 mg standard into 10 mL of acetonitrile: water: orthophosphoric acid (50:49.9:0.1 *V*/*V*/*V*) - diluent 2 and diluting the obtained stock solution 1000-fold (c_{sample} = 0.001 mg/mL).

Naringenin, piperine, and aniracetam reference solutions were prepared by dissolving 10 mg of standards into a 10 mL of diluent 2 and diluting the obtained stock solutions 100-fold (c_{sample} = 0.01 mg/mL).

Piperyline reference solution was prepared by dissolving 5 mg standard into 10 mL of diluent 2 and diluting the obtained stock solutions 100-fold (c_{sample} = 0.005 mg/mL).

For L-theanine the chromatographic analysis was performed on the Apollo C_{18} column (4.6 mm × 150 mm, 5 μm; Alltech, Lokeren, Belgium). Column temperature was maintained at 40°C with a flow rate of 1 mL/min. For the mobile phase, a mixture of acetonitrile (eluent B) and 0.1M phosphate buffer (pH 2.5) with 0.015M heptane-1-sulfonic acid sodium salt (eluent A) was used. The linear gradient mode used for the elution was as follows: 0-7 min, 5%-60% B; 8-9 min, 60%-5% B; 10-12 min, 5% B. The UV detection was performed at 210 nm and injection volume was set to 25 μL.

L-Theanine reference solution was prepared by dissolving 20 mg standard into 10 mL of water and diluting the obtained stock solution 100-fold (c_{sample} = 0.02 mg/mL).

The equivalent to the weight of 1 capsule (Table 1 in the main text) of each online purchased phenibut product was extracted in 100 mL of the appropriate diluent (in triplicate) with the aid of sonication for 10 minutes at 40°C to obtain respective stock solutions. The suspensions were cooled to room temperature, filtered through a PTFE filter with a pore diameter of 0.45 μm (the resulting filtrates were used for the HPLC assay of SAM, Aniracetam, Huperzine A) and diluted ten-fold, resulting in

analytical sample solutions that were used for the HPLC assays of L-DOPA, 5-HTP, naringenin, naringin, piperine, piperyline and L-theanine.

Other added ingredient content in online purchased phenibut product capsules was determined by plotting the mean peak area of the appropriate compound in analytical sample solutions against the mean peak area of the compound in reference solutions. The amount of naringin was calculated by plotting the mean peak area of naringin against naringenin standard with 0.01 mg/mL concentration due to identical extinction coefficients in the 280 nm range [1].

2. Tables

Table S1. Online purchased phenibut product capsule excipients.

#	Content	Sample 1 (NEXTVALLEY, Phenibut HCl)	Sample 2 (PARADIGM Peptides, Phenibut)	Sample 3 (CORE LABS X, Pheni-B Ultra)	Sample 4 (REVANGE nutrition, Phenibut)	Sample 5 (REVANGE nutrition, Phenibut^{RX})	Sample 6 (REVANGE nutrition, Pheni+)
**1**	Gluten-free organic rice flour	x					
**2**	Magnesium stearate	x		x	x	x	x
**3**	Hydroxypropyl-methylcellulose	x					
**4**	Vegetable gelatin		x				
**5**	Vegetable magnesium stearate		x				
**6**	Silicon dioxide			x	x	x	x

Table S2. The manufacturer-provided information on the use of online purchased phenibut products.

#	Supplier	Name	API labelled (mg)	Suggested use (as labelled on the packaging or from the manufacturer web page)	Corresponding Phenibut dosage (mg)	Dossage corresponding to API_{found} (mg)	Comments
1	NEXTVALLEY	Phenibut HCl	500	Because the nootropic synthetic substance is not intended for human consumption, it is questionable to mention information that is clearly about consuming the substance. Where certain information is desired or even mandatory for food supplements, no notification is made for these substances because it may then be possible to interpret that the substance is intended for human consumption[a].	*n/a*	*n/a*	
2	PARADIGM peptides	PHENIBUT	250	When it comes to Phenibut dosage, there is not a specific amount for everyone. It depends on several factors, such as the person's age and overall health. Of course, while there is no particular Phenibut dose, there are some guidelines.<br>For example, it is recommended that you start with a 250mg/day Phenibut dosage when starting and gradually build up to 1-2 grams per day. The Phenibut nootropic can be habit-forming if taken in excess. With that in mind, correct usage and proper Phenibut dosages are strongly advised. If you build up a high tolerance and suddenly stop taking the nootropic, Phenibut withdrawal can occur[b].	*n/a*	*n/a*	

(*continued on next page*)

Table S2 (*continued*)

3	CORE LABS X	PHENI-B ULTRA	500	Take 3 × 1 capsule daily or 2-3 capsules at once preferably in the evening, depending on your goal and needs). Do not exceed recommended daily intake[c].	1500	1572	corresponds to the daily recommended 3 × 1 capsule intake
					1000 - 1500	1048 - 1572	corresponds to the recommended 2-3 capsule intake at once
4	REVANGE nutrition	PHENIBUT	500	As a dietary supplement, take 1-2 capsules 2-4 times per day. To promote restful sleep, consume 2-6 capsules before bed. Start at the lower doe to assess tolerance. Do not take for more than 3 days in a row without a 2-3 day wash out. Use only as directed. Do not exceed recommended daily intake[c].	1000 - 4000	770 - 3080	corresponds to the daily minimum recommended (1 capsule 2 times per day) - daily recommended maximum (2 capsules 4 times per day).
					1000 - 3000	770 - 2310	corresponds to minimum recommended (2) - maximum recommended (6) capsule intake at once (before sleep).
5	REVANGE nutrition	PHENIBUT RX	900	As a dietary supplement, take 1 capsule 2-4 times per day. To promote restful sleep, consume 1-3 capsules before bed. Start at the lower dose to assess tolerance. Do not take for more than 3 days in a row without a 2-3 day wash out. Use only as directed. Do not exceed recommended daily intake[c].	1800 - 3600	1378 - 2756	corresponds to the daily minimum recommended (1 capsule 2 times per day) - daily recommended maximum (1 capsule 4 times per day).
					900-2700	770-2067	corresponds to the minimum recommended (1) - maximum recommended (3) capsule intake at once (before sleep).

(*continued on next page*)

Table S2 (*continued*)

6	REVANGE nutrition	PHENI+	500	Take 3 × 1 capsule daily or 2-3 capsules at once preferably in the evening, depending on your goal and needs). Do not exceed recommended daily intake[c].	1500	2004	corresponds to the daily minimum recommended (1 tablet 3 times per day) - daily recommended maximum (2 tablets 3 times per day).
					1000-1500	1336-2004	corresponds to the maximum recommended 3-capsule intake at once

[a] https://nextvalley.com/what-is-the-legal-status-of-a-nootropic/

[b] https://paradigmpeptides.com/product/phenibut/

[c] Daily value not established for any of the compounds stated on packaging

3. Figures

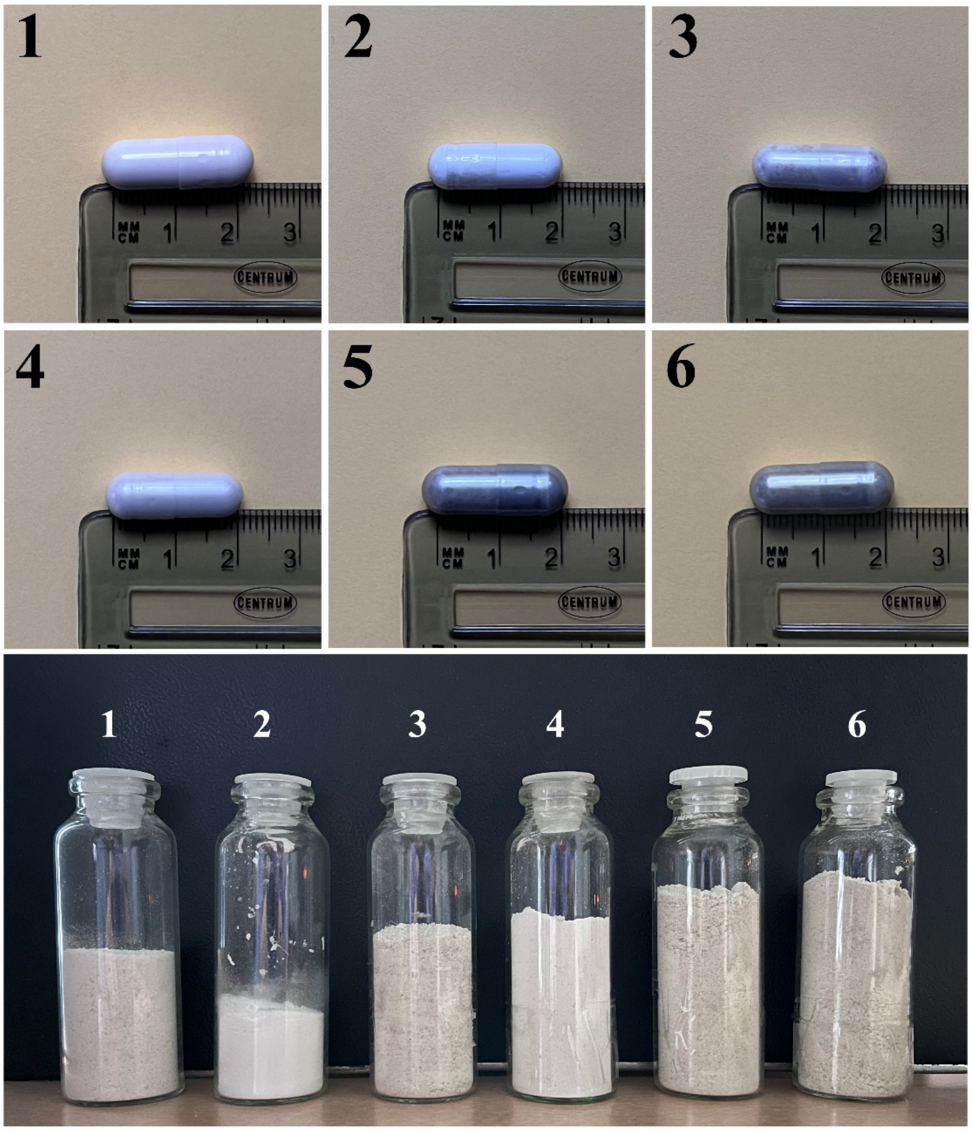

Figure S1. Visual inspection of online purchased phenibut products.

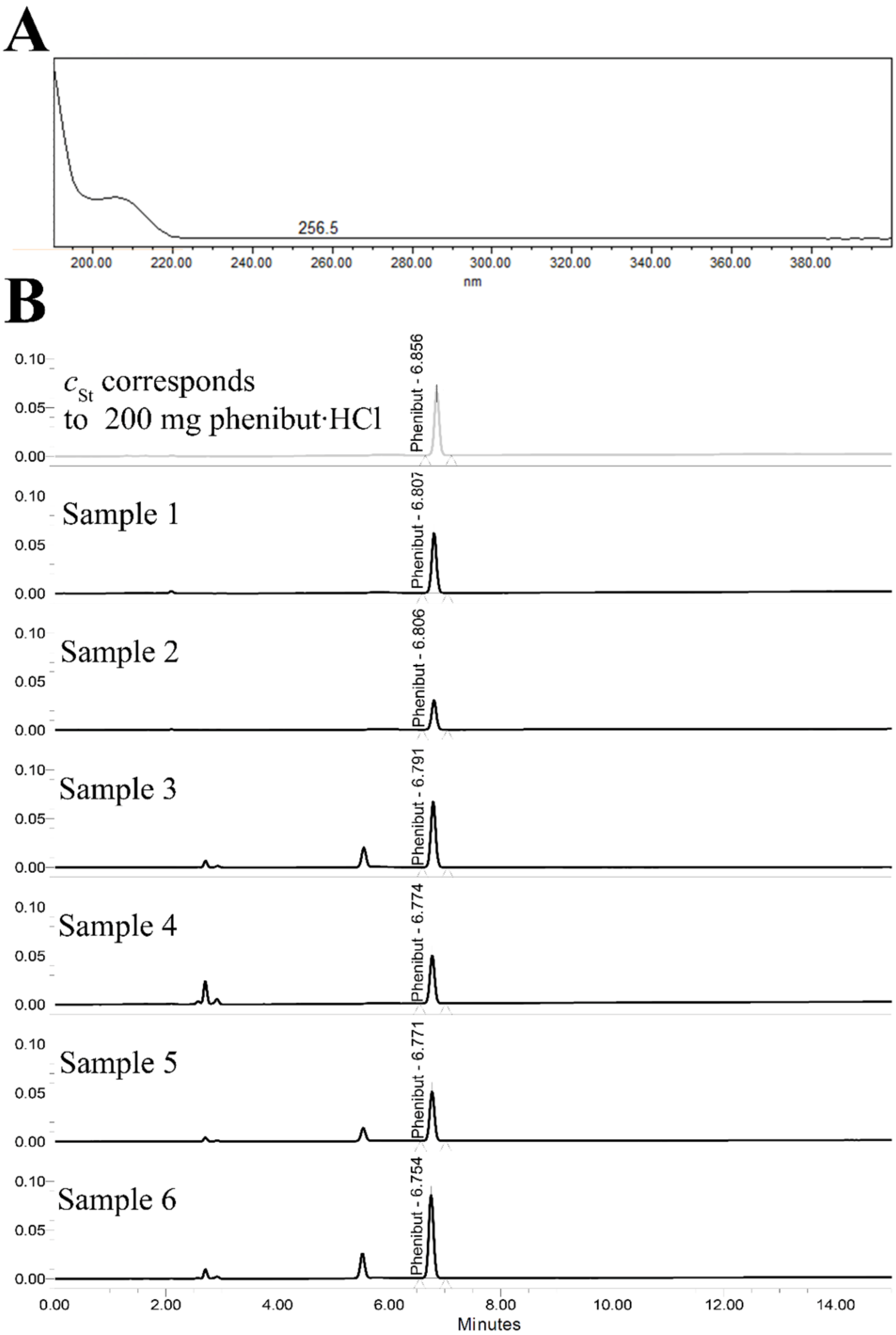

Figure S2. HPLC-DAD analysis of phenibut contents in online purchased products. UV spectra of phenibut (A). Representative HPLC plots of phenibut·HCl reference standard (*in grey*) and samples obtained from products **1 – 6** (*in black*) (B). Column: YMC-pack C_8 (4.6 mm × 150 mm, 5 µm); mobile phase: acetonitrile with 0.02M phosphate buffer (pH 2.8) in water (2:98 *V/V*; eluent A) and acetonitrile with 0.02M phosphate buffer (pH 2.8) in water (70:30 *V/V*; eluent B); Gradient elution mode: 0-25 min, 0%-50% B; 25-30 min, 50% B; 30-33 min, 50%-0% B; 33-40 min 0% B; Flow rate: 1 mL/min; Column temperature: 30ºC; UV detection: λ = 210 nm.

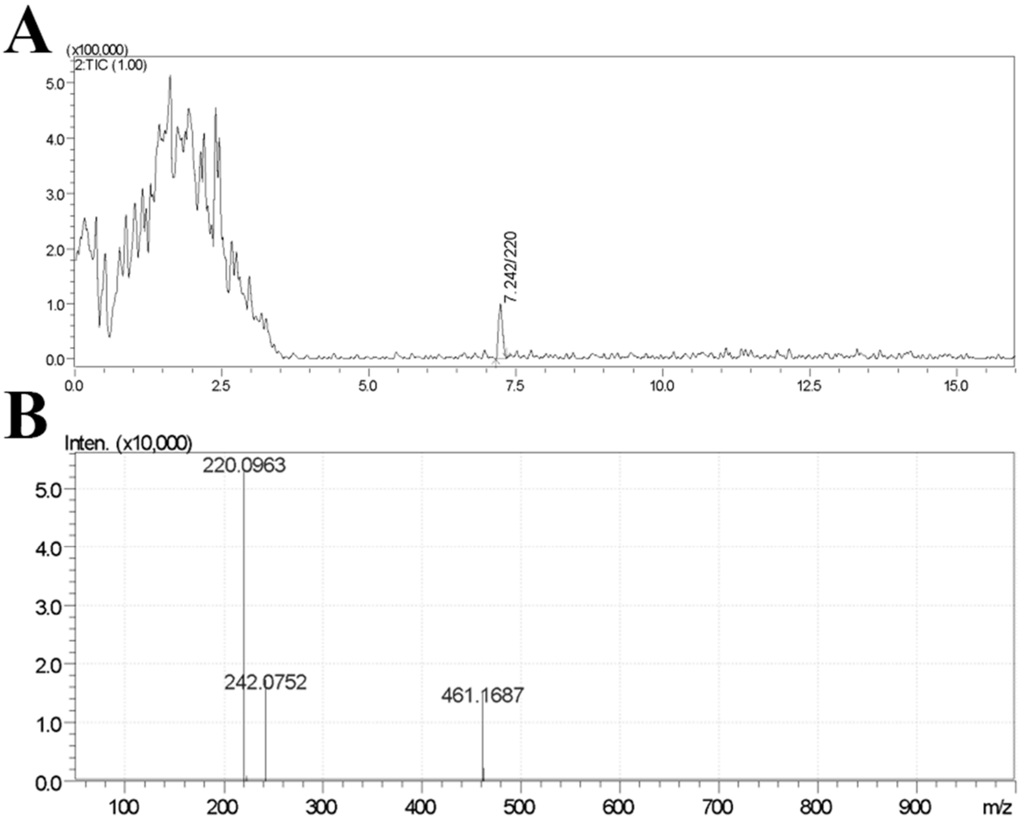

Figure S3. UHPLC-TOF-MS analysis of sample **1**. Total ion chromatogram (A). No other ingredients were specified for this phenibut product. TOF-MS spectrum of peak at RT 7.24 min of sample **1**, tentatively identified as aniracetam (B): m/z 220 $[M+H]^+$; m/z 242 $[M+Na]^+$; m/z 461 $[2M+Na]^+$.

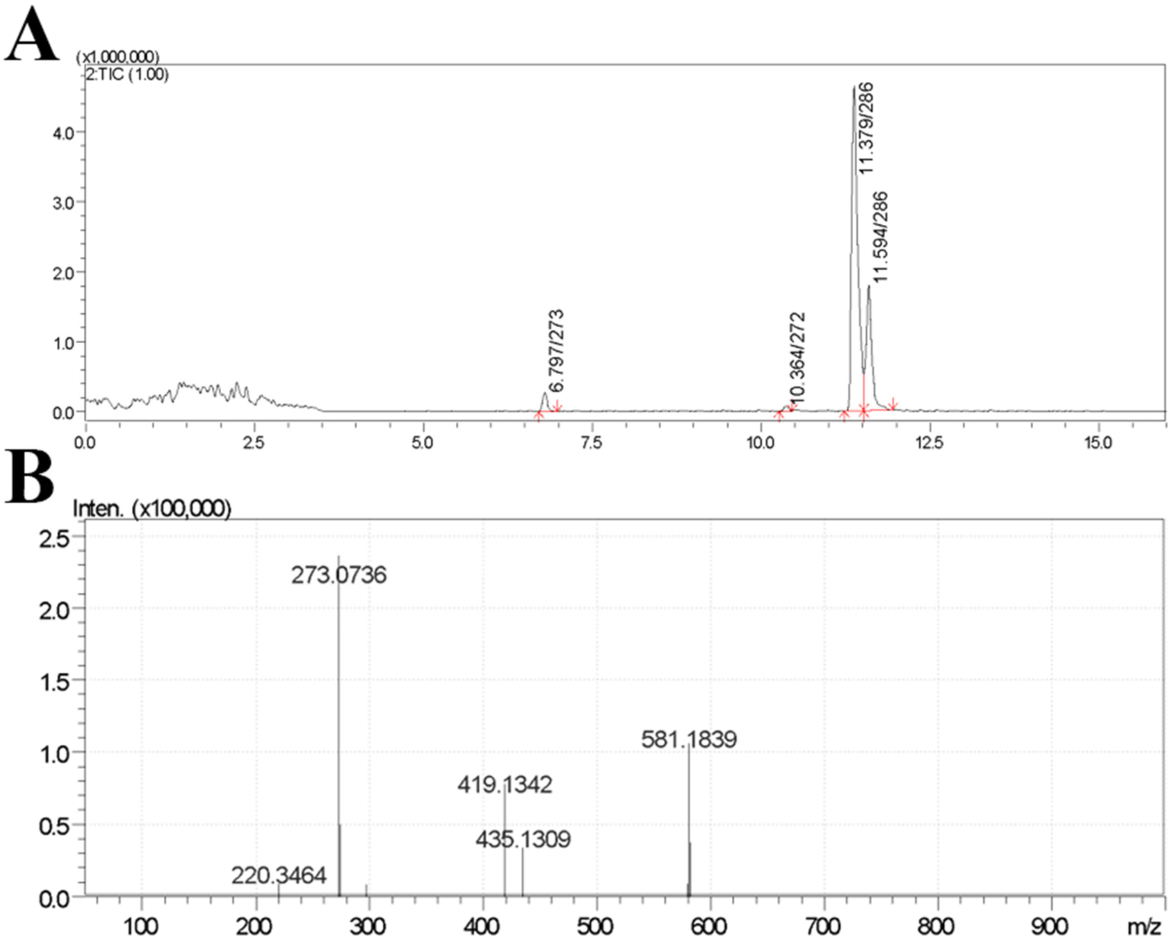

Figure S4. UHPLC-TOF-MS analysis of sample **3**. Total ion chromatogram (A). Peaks at RT 6.797 min and 10.364 min do not correspond to any of the declared ingredients. The peak at RT 11.379 min corresponds to piperine which is declared in sample **6** only. TOF-MS spectrum of peak at RT 6.80 min of sample **3**, tentatively identified as naringin (B).

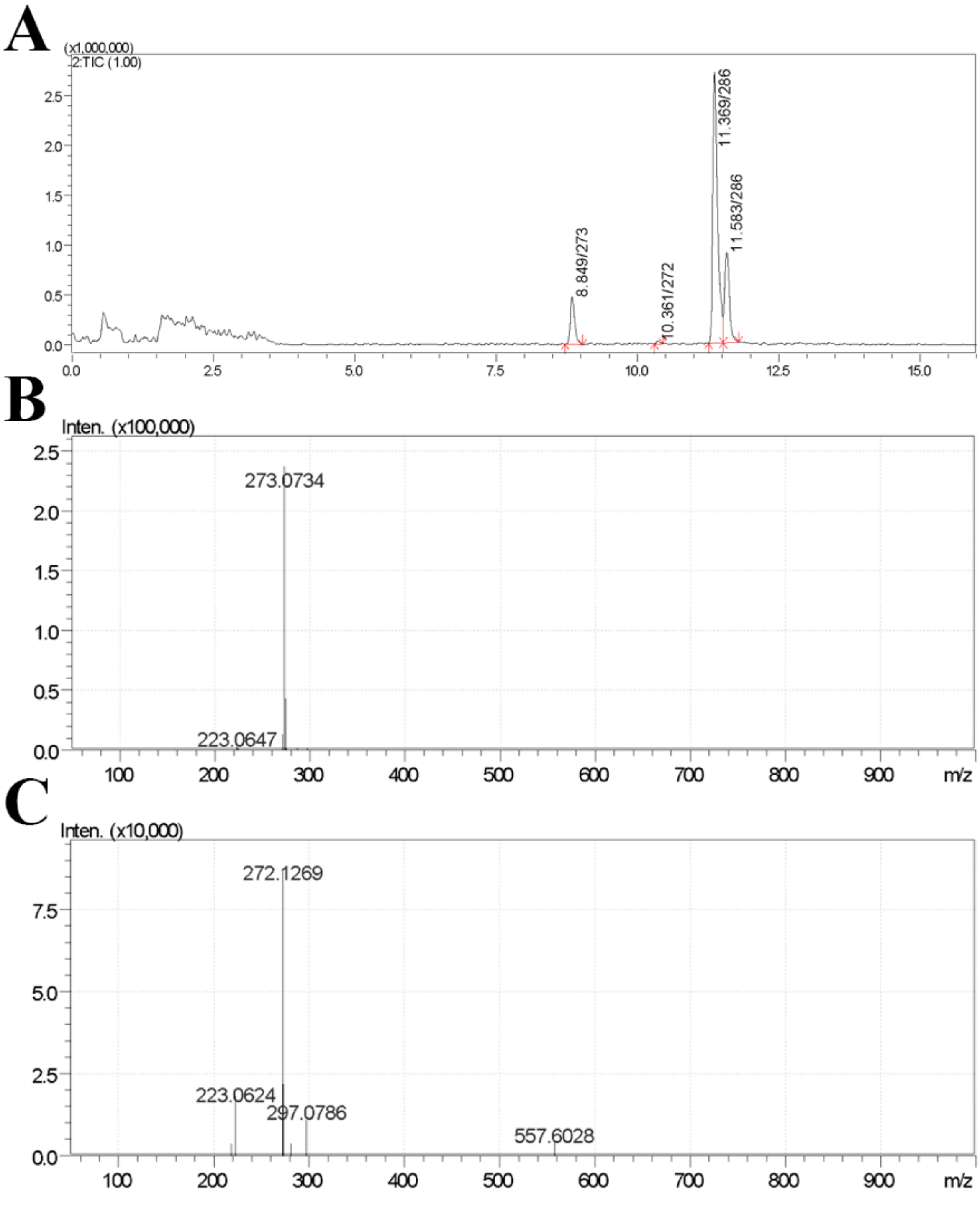

Figure S5. UHPLC-TOF-MS analysis of sample **4**. Total ion chromatogram (A). The peak at RT 8.849 min corresponds to naringenin. TOF-MS spectrum of chromatographic peak at RT 8.849 min in phenibut product **4**. Obtained spectrum corresponds to naringenin (Δ m/z 2.4 mDa) (B). TOF-MS spectrum of peak at RT 10.36 min of sample **4**, tentatively identified as piperyline (C).

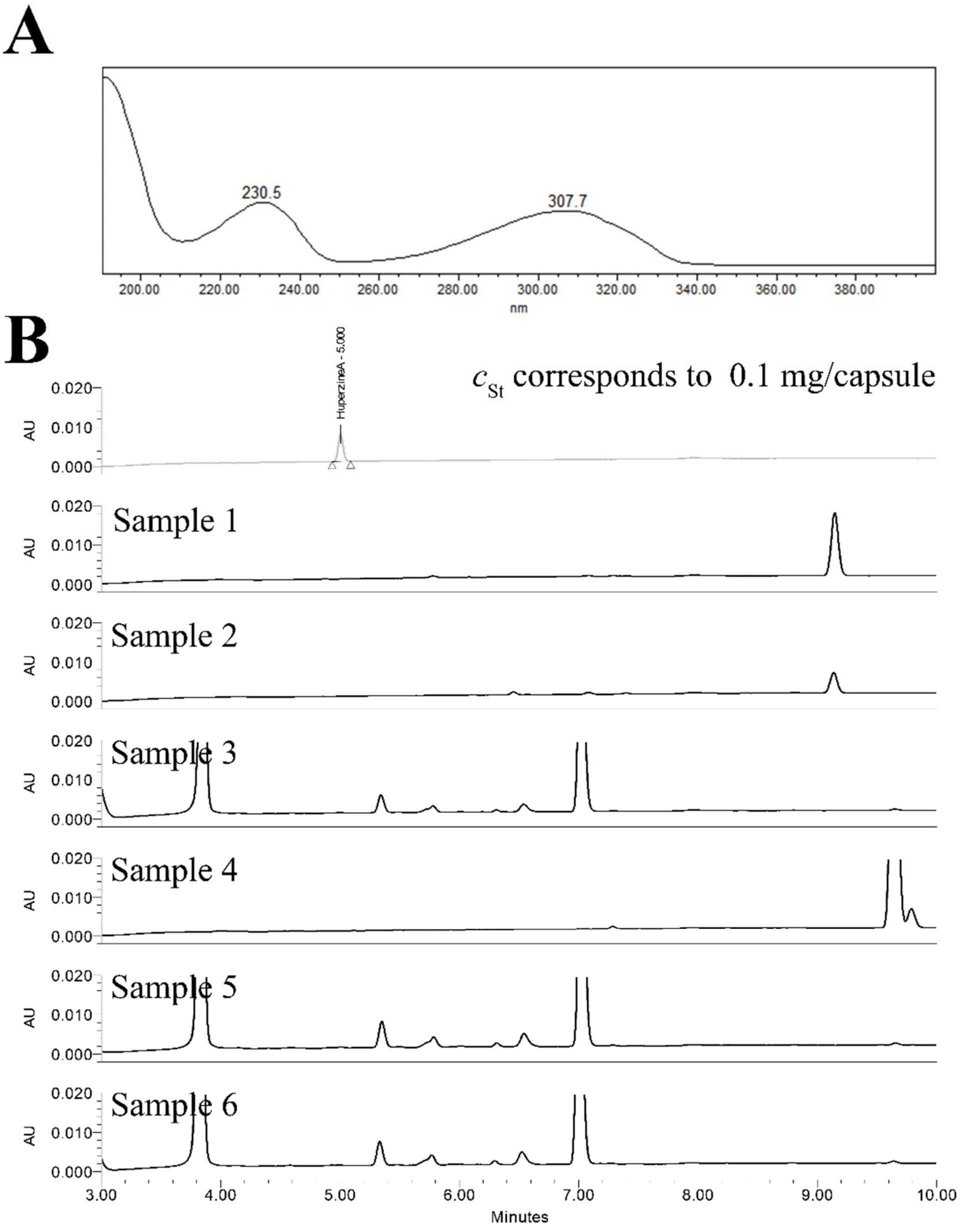

Figure S6. Huperzine A HPLC-DAD analysis in online purchased phenibut products. UV spectra of huperzine A (A). Representative HPLC plots of huperzine A reference standard (*in grey*) and samples obtained from products **1** – **6** (*in black*) (B). Column: Apollo C_{18} (4.6 mm × 150 mm, 5 μm); mobile phase: acetonitrile (eluent B) with 0.1% orthophosphoric acid in water (eluent A); Gradient elution mode: 0-15 min, 5%-95% B; 15-20 min, 95% B; 20-22 min, 95%-5% B; 22-25 min 5% B; Flow rate: 1 mL/min; Column temperature: 40ºC; UV detection: λ = 308 nm.

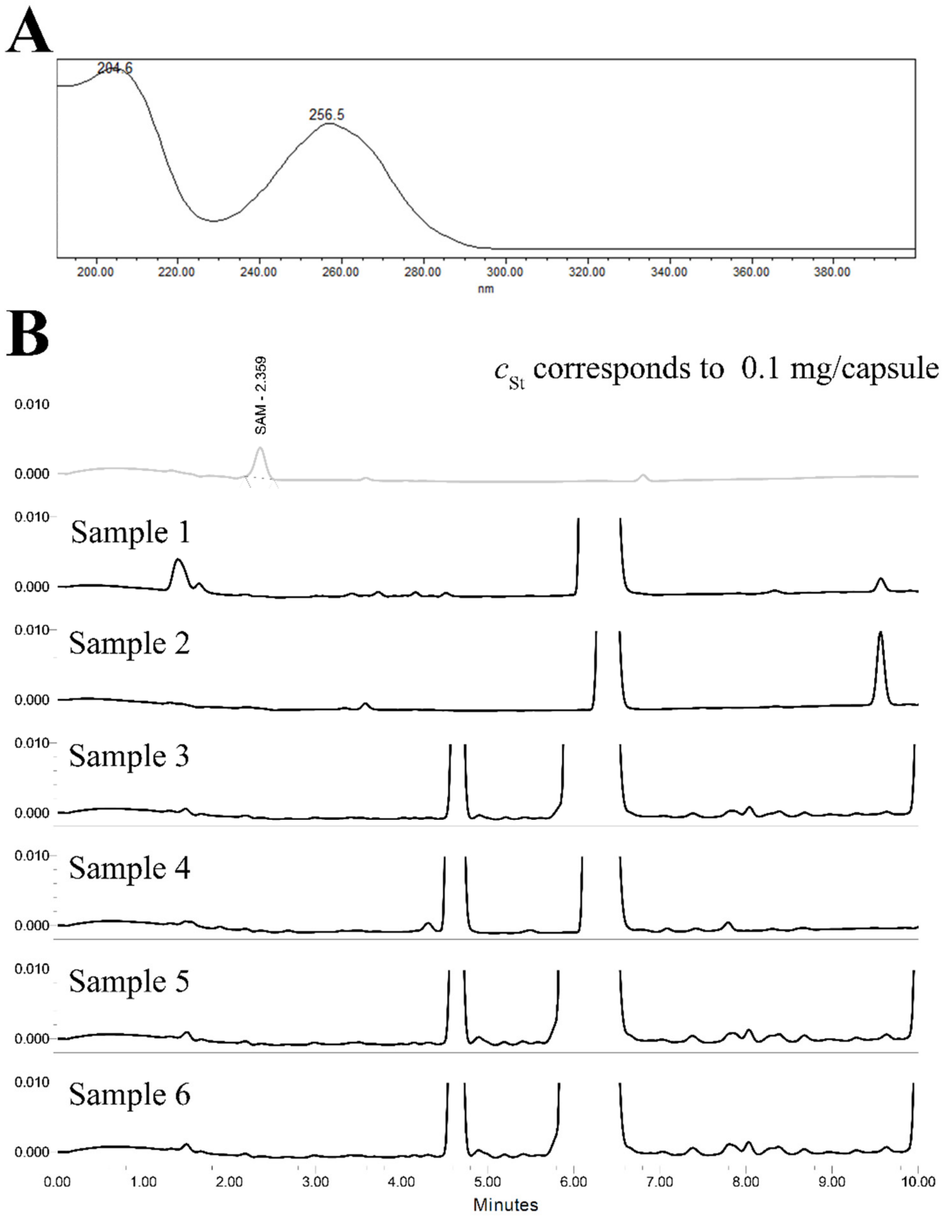

Figure S7. *S*-(5'-Adenosyl)-L-methionine (SAM) HPLC-DAD analysis in online purchased phenibut products. UV spectra of SAM (A). Representative HPLC plots of SAM reference standard (*in grey*) and samples obtained from products **1** – **6** (*in black*) (B). Column: Apollo C_{18} (4.6 mm × 150 mm, 5 µm); mobile phase: methanol (eluent B) with 0.2% trifluoroacetic acid in water (eluent A); Gradient elution mode: 0-15 min, 5%-95% B; 15-20 min, 95% B; 20-22 min, 95%-5% B; 22-25 min 5% B; Flow rate: 1 mL/min; Column temperature: 40ºC; UV detection: λ = 254 nm.

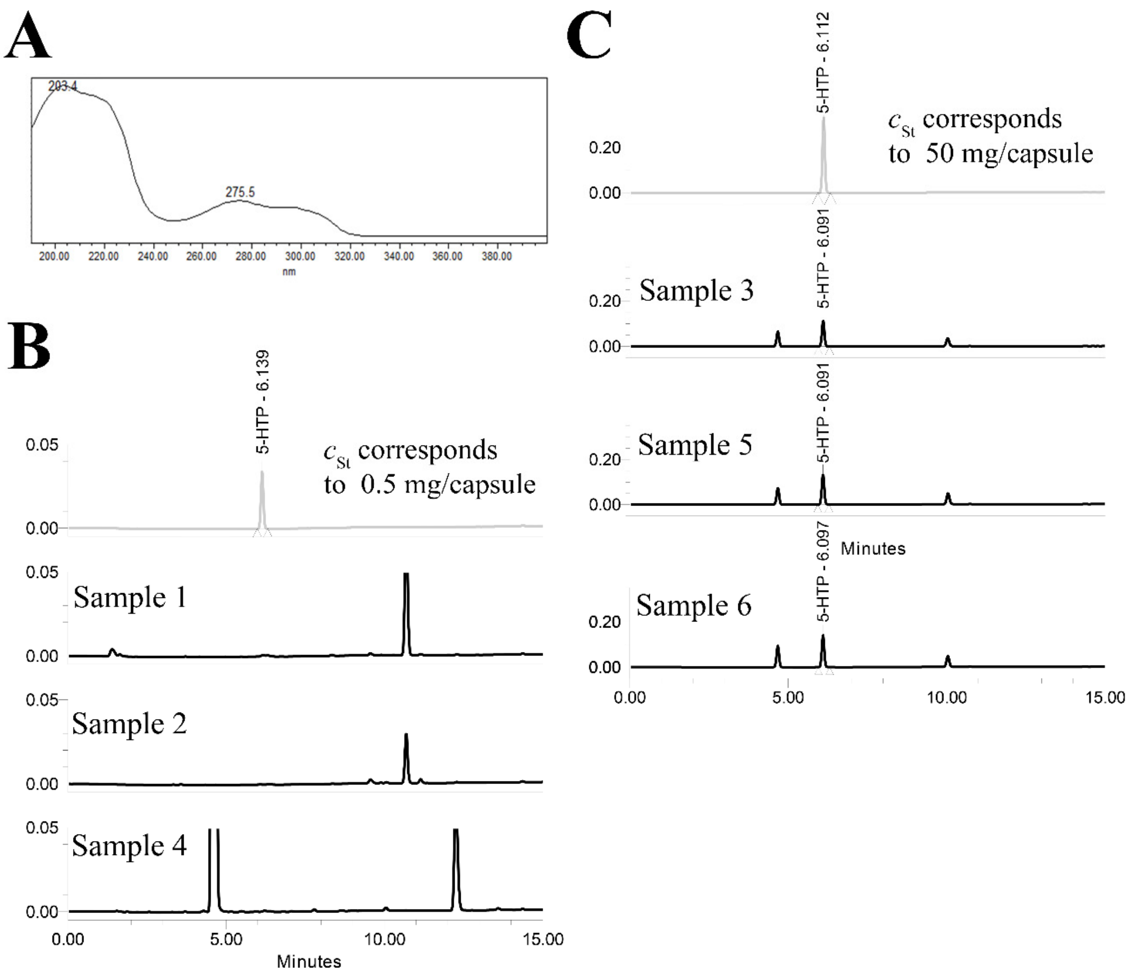

Figure S8. 5-Hydroxy-L-tryptophan (5-HTP) HPLC-DAD analysis in online purchased phenibut products. UV spectra of 5-HTP (A). Representative HPLC plots of 5-HTP sensitivity standard (*in grey*) and samples obtained from products **1**, **2,** and **4** (*in black*) (B). Representative HPLC plots of 5-HTP reference standard (*in grey*) and samples obtained from products **3**, **5,** and **6** (*in black*) (C). Column: Apollo C_{18} (4.6 mm × 150 mm, 5 µm); mobile phase: methanol (eluent B) with 0.2% trifluoroacetic acid in water (eluent A); Gradient elution mode: 0-15 min, 5%-95% B; 15-20 min, 95% B; 20-22 min, 95%-5% B; 22-25 min 5% B; Flow rate: 1 mL/min; Column temperature: 40ºC; UV detection: λ = 275 nm.

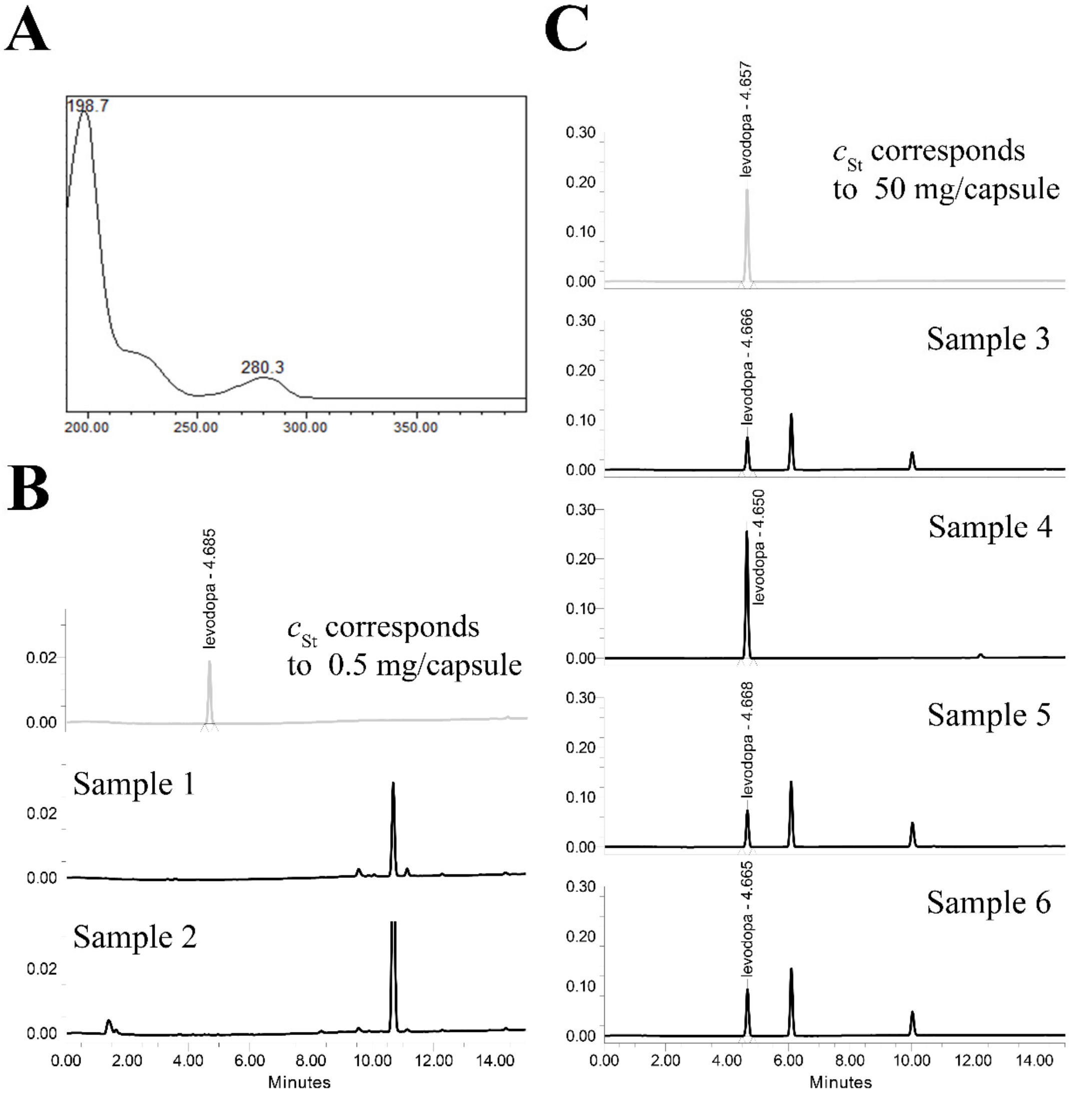

Figure S9. L-3,4-Dihydroxyphenylalanine (L-DOPA) HPLC-DAD analysis in online purchased phenibut products. UV spectra of L-DOPA (A). Representative HPLC plots of L-DOPA sensitivity standard (*in grey*) and samples obtained from products **1** - **2** (*in black*) (B). Representative HPLC plots of L-DOPA reference standard (*in grey*) and samples obtained from products **3** - **6** (*in black*) (C). Column: Apollo C_{18} (4.6 mm × 150 mm, 5 μm); mobile phase: methanol (eluent B) with 0.2% trifluoroacetic acid in water (eluent A); Gradient elution mode: 0-15 min, 5%-95% B; 15-20 min, 95% B; 20-22 min, 95%-5% B; 22-25 min 5% B; Flow rate: 1 mL/min; Column temperature: 40ºC; UV detection: λ = 275 nm.

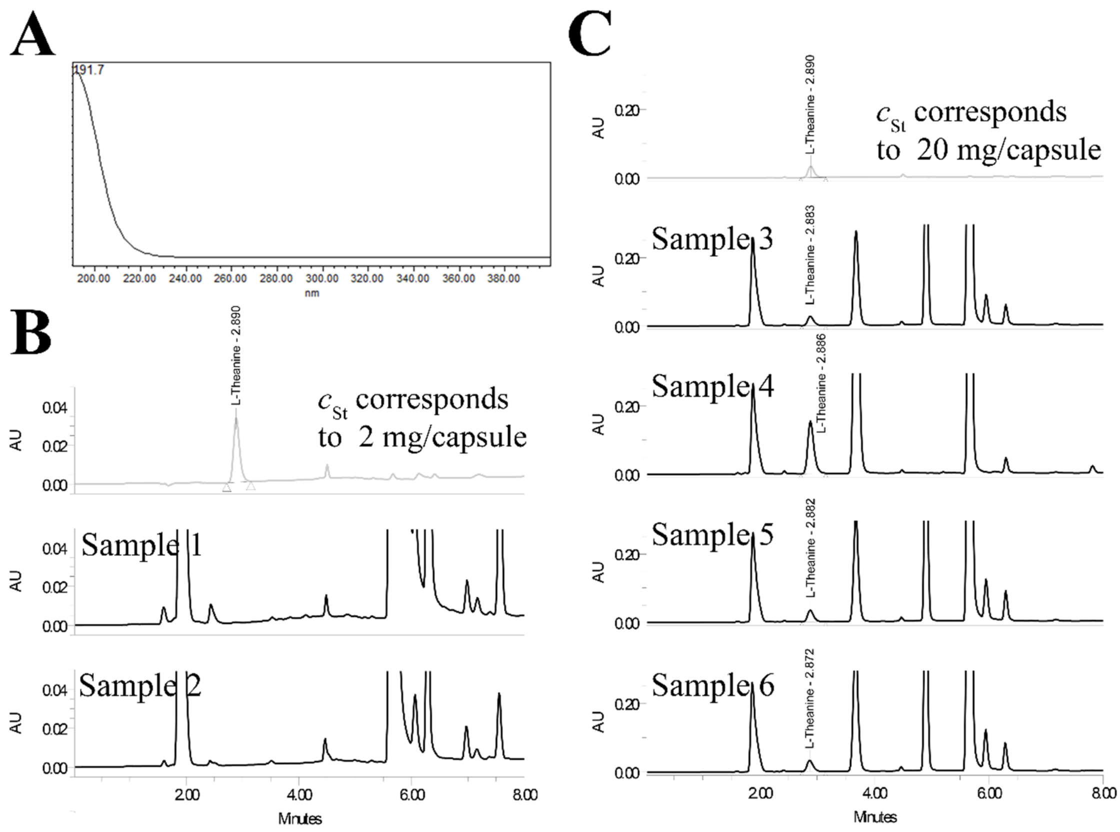

Figure S10. L-Theanine HPLC-DAD analysis in online purchased phenibut products. UV spectra of L-theanine (A). Representative HPLC plots of L-theanine sensitivity standard (*in grey*) and samples obtained from products **1** - **2** (*in black*) (B). Representative HPLC plots of L-theanine reference standard (*in grey*) and samples obtained from products **3** - **6** (*in black*) (C). Column: Apollo C_{18} (4.6 mm × 150 mm, 5 µm); mobile phase: acetonitrile (eluent B) and 0.1M phosphate buffer (pH 2.5) with 0.015M heptane-1-sulfonic acid sodium salt (eluent A); Gradient elution mode: 0-7 min, 5%-60% B; 8-9 min, 60%-5% B; 10-12 min 5% B; Flow rate: 1 mL/min; Column temperature: 40ºC; UV detection: λ = 210 nm.

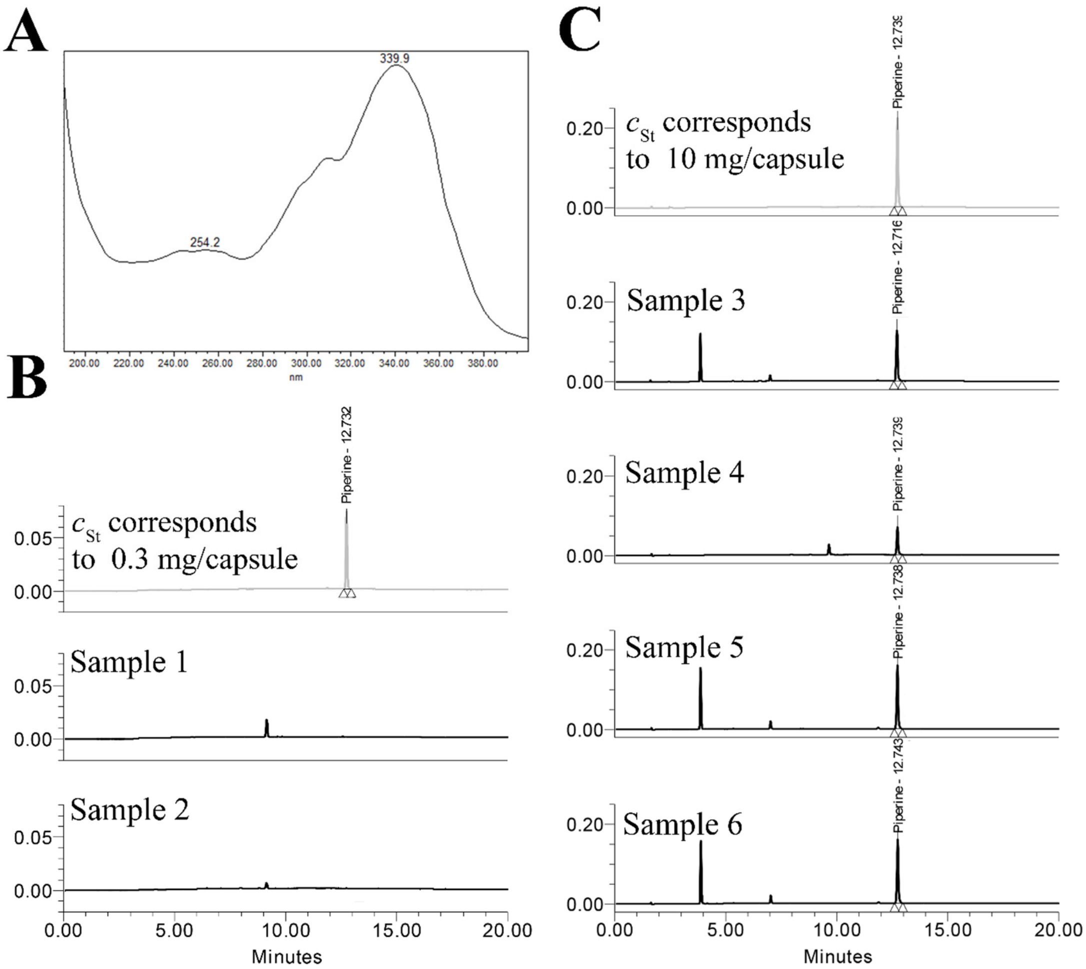

Figure S11. Piperine HPLC-DAD analysis in online purchased phenibut products. UV spectra of piperine (A). Representative HPLC plots of piperine sensitivity standard (*in grey*) and samples obtained from products **1** - **2** (*in black*) (B). Representative HPLC plots of piperine reference standard (*in grey*) and samples obtained from products **3** - **6** (*in black*) (C). Column: Apollo C_{18} (4.6 mm × 150 mm, 5 µm); mobile phase: acetonitrile (eluent B) with 0.1% orthophosphoric acid in water (eluent A); Gradient elution mode: 0-15 min, 5%-95% B; 15-20 min, 95% B; 20-22 min, 95%-5% B; 22-25 min 5% B; Flow rate: 1 mL/min; Column temperature: 40ºC; UV detection: λ = 308 nm.

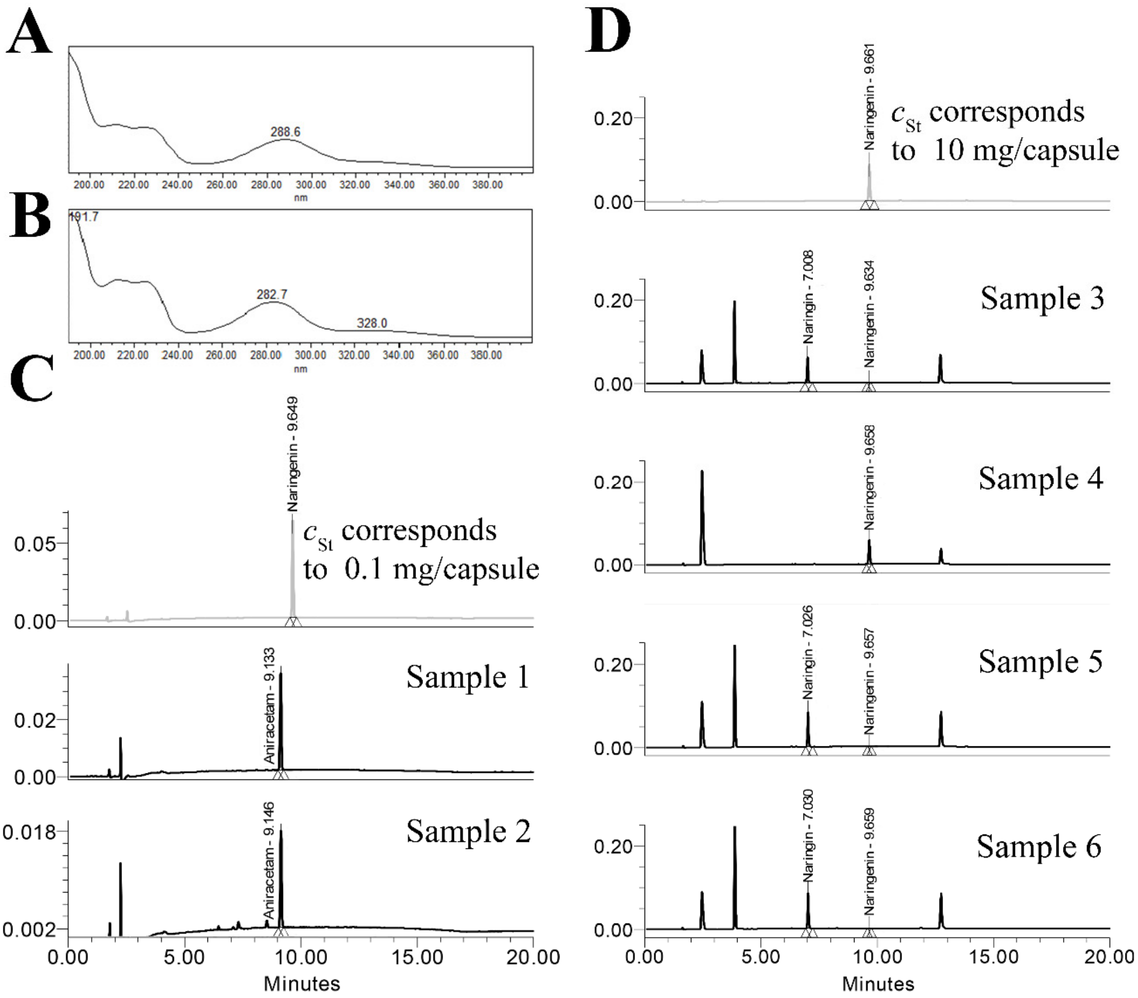

Figure S12. Naringenin and naringin HPLC-DAD analysis in online purchased phenibut products. UV spectra of naringenin (A). UV spectra of naringin (B). Representative HPLC plots of naringenin sensitivity standard (*in grey*) and samples obtained from products **1** - **2** (*in black*) (C). Representative HPLC plots of naringenin reference standard (*in grey*) and samples obtained from products **3** - **6** (*in black*). Column: Apollo C_{18} (4.6 mm × 150 mm, 5 µm); mobile phase: acetonitrile (eluent B) with 0.1% orthophosphoric acid in water (eluent A); Gradient elution mode: 0-15 min, 5%-95% B; 15-20 min, 95% B; 20-22 min, 95%-5% B; 22-25 min 5% B. Flow rate: 1 mL/min; Column temperature: 40ºC; UV detection: λ = 280 nm.

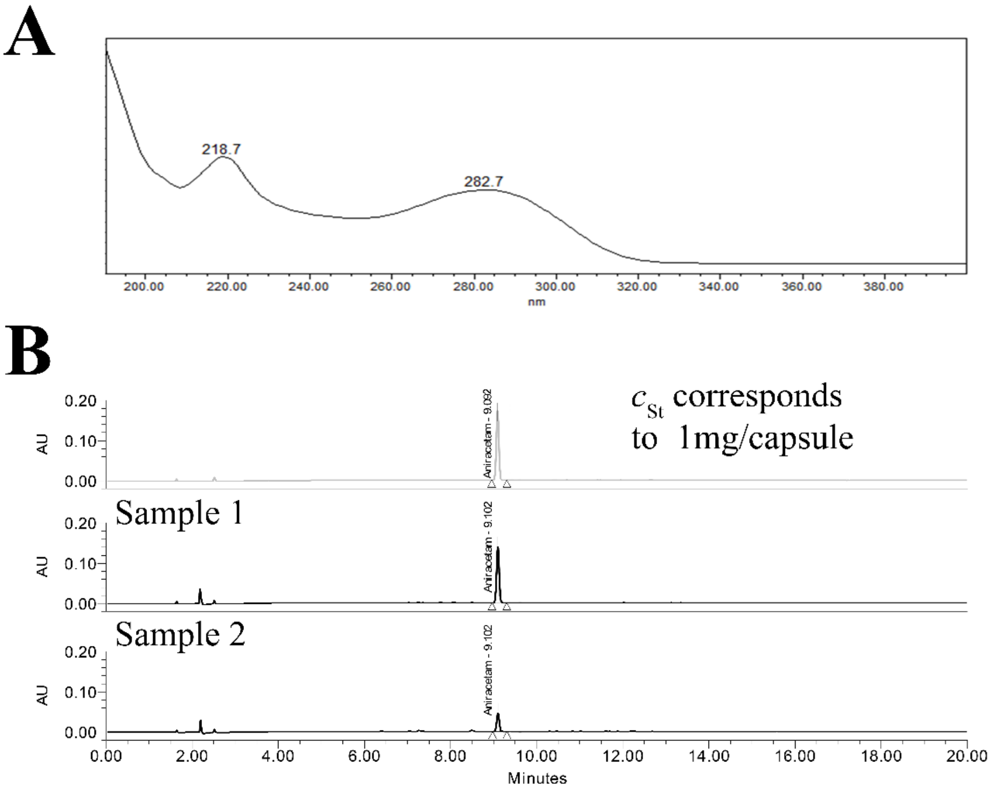

Figure S13. Aniracetam HPLC-DAD analysis in online purchased phenibut products. UV spectra of aniracetam (A). Representative HPLC plots of aniracetam reference standard (*in grey*) and samples obtained from products **1** and **2** (*in black*) (B). Column: Apollo C_{18} (4.6 mm × 150 mm, 5 µm); mobile phase: acetonitrile (eluent B) with 0.1% orthophosphoric acid in water (eluent A); Gradient elution mode: 0-15 min, 5%-95% B; 15-20 min, 95% B; 20-22 min, 95%-5% B; 22-25 min 5% B. Flow rate: 1 mL/min; Column temperature: 40ºC; UV detection: λ = 280 nm.

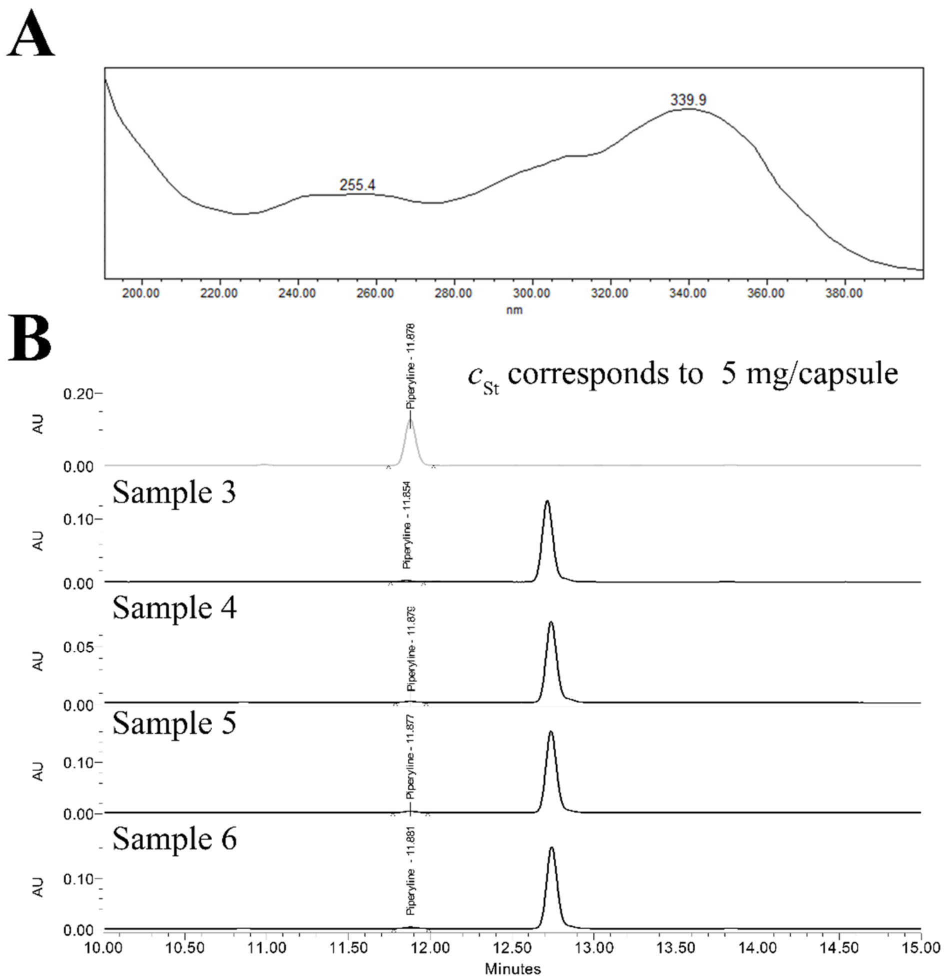

Figure S14. Piperyline HPLC-DAD analysis in online purchased phenibut products. UV spectra of piperyline (A). Representative HPLC plots of piperyline reference standard (*in grey*) and samples obtained from products **3** - **6** (*in black*) (B). Column: Apollo C_{18} (4.6 mm × 150 mm, 5 µm); mobile phase: acetonitrile (eluent B) with 0.1% orthophosphoric acid in water (eluent A); Gradient elution mode: 0-15 min, 5%-95% B; 15-20 min, 95% B; 20-22 min, 95%-5% B; 22-25 min 5% B. Flow rate: 1 mL/min; Column temperature: 40ºC; UV detection: λ = 308 nm.

References

[1] LM Cordenonsi, RM Sponchiado, SC Campanharo, CV Garcia, RP Raffin, EES Schapoval. Study of Flavonoids presente in Pomelo (Citrus máxima) by DSC, UV-VIS, IR, 1 H AND 13 C NMR AND MS [J]. Drug Analytical Research, 2017, 01: 31-37.
